# What is the current status of research for esketamine in the treatment of bipolar depression? A literature review

**DOI:** 10.1192/j.eurpsy.2026.11764

**Published:** 2026-07-31

**Authors:** O. Vasiliu, D. G. Vasiliu

**Affiliations:** 1 Clinical Neurosciences Department, “Carol Davila” University of Medicine and Pharmacy; 2 Psychiatry Department, “Dr. Carol Davila” University Emergency Central Military Hospital; 3 White Yellow Cross Foundation, Bucharest, Romania

## Abstract

**Introduction:**

Hopes related to esketamine have been raised by its approval for treatment-resistant major depression (TRD) in the sense that this antidepressant, with a novel mechanism of action, will be useful for patients with bipolar depression (BD), too. The need to find more evidence-based pharmacological approaches to both TRD and BD is acute due to the high rates of partial response or non-responsiveness in these conditions.

**Objectives:**

The aim of this review was to gather the evidence in the literature regarding the effects of esketamine in patients diagnosed with BD.

**Methods:**

A literature review was conducted in three electronic databases (PubMed, EMBASE, and Clarivate/Web of Science) using the keywords „bipolar disorder,” „esketamine,” and „glutamatergic antidepressants” for papers published in English between January 2000 and February 2025.

**Results:**

Seven primary and secondary reports were identified and further investigated. Data regarding BD is difficult to separate from TRD because cases are usually reported together in clinical trials, which makes it challenging to extract valid conclusions about BD. Only data from case reports (N=2) show positive results and good tolerability of esketamine intranasal spray in combination with mood stabilizers. Data in clinical trials exploring ketamine and subcutaneous or intravenous esketamine support the favorable effects of the active drugs vs. placebo in both TRD and BD. Further studies with a good-quality, homogenous population and long-term monitoring of patients with BD are needed to support the utility of esketamine.

**Conclusions:**

Due to its unique mechanism of action in the currently approved spectrum of antidepressants, esketamine raises expectations about its potential efficacy for BD that need to be explored in future studies.

**Disclosure of Interest:**

None Declared

